# Targeted molecular docking-guided insights into the antioxidant reparative mechanisms of *Brassica rapa L.* extract after H_2_O_2_-induced neurotoxicity in PC-12 cells: Integration of LC-MS metabolomics and transcriptomic responses

**DOI:** 10.1016/j.bbrep.2026.102566

**Published:** 2026-03-30

**Authors:** Wenyuan Wan, Yuntao Zhang, Xiaotong Yang, Jinyao Li, Jun Lu, Yu Zhao

**Affiliations:** a College of Life, Shanghai Normal University, 100 Guilin Road, Shanghai, 200234, China; b Xinjiang Key Laboratory of Biological Resources and Genetic Engineering, College of Life Science and Technology, Xinjiang University, Urumqi, 830046, China; c Auckland Bioengineering Institute, University of Auckland, Auckland, 1142, New Zealand; d School of Chemical Sciences, University of Auckland, Auckland, 1142, New Zealand; e Department of Food and Agriculture Technology, Yangtze Delta Region Institute of Tsinghua University, Jiaxing, Zhejiang, 314006, China

**Keywords:** *Brassica rapa L.*, LC-MS, Neuroprotection, Transcriptomics, Molecular docking, Antioxidant pathways

## Abstract

This study investigated the reparative effects of *Brassica rapa L.* ethanolic extract (BREE) on H_2_O_2_-induced oxidative injury in PC-12 cells. *Brassica rapa L.*, a cruciferous plant rich in bioactive flavonoids, exhibits potent antioxidant and neuroprotective properties against oxidative stress, a key pathological driver in neurodegenerative diseases. This study investigated the reparative effects of *Brassica rapa L.* ethanolic extract (BREE) on H_2_O_2_-induced oxidative injury in PC-12 cells, employing a multi-omics approach integrating LC-MS/MS metabolite profiling, molecular docking, and transcriptomic validation. BREE demonstrated robust radical-scavenging activity. Molecular docking identified direct interactions among flavonoids within BREE, suggesting a role in redox modulation. Subsequent experiments confirmed the activation of the PI3K/Akt, Keap1-Nrf2, and cell cycle pathways. Functionally, BREE significantly restored cell viability to 162.3% ± 6.9% of that in the control group. These findings validate BREE's dual action in scavenging ROS and reprogramming redox signaling networks, positioning it as a candidate for combating oxidative stress-associated neurodegeneration and as a potential functional food for age-related cognitive decline.

## Highlights

• Integrating LC-MS metabolomics, transcriptomics, and molecular docking to study bioactives.
• Revealed BREE's dual action in scavenging ROS and reprogramming redox signaling pathways.
• We validated BREE's function to restored H_2_O_2_-injured PC-12 cell viability.
• Findings position BREE as a candidate for mitigating age-related cognitive decline.

## Graphical abstract

**Figure:**
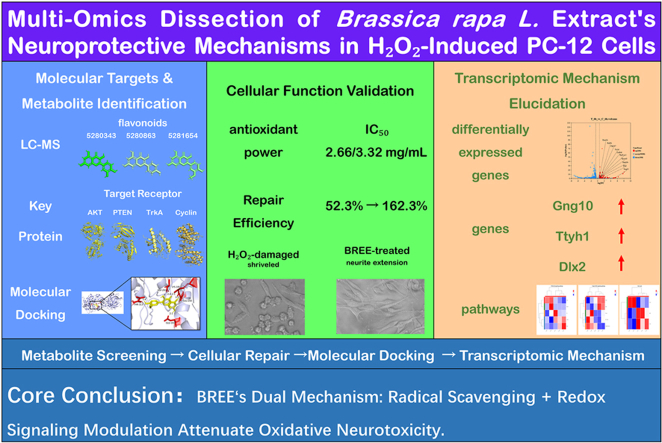

## 1 Introduction

Oxidative stress constitutes a fundamental biological mechanism underlying both homeostatic regulation and pathological progression, primarily stemming from overproduction of reactive oxygen species (ROS)[1,2]. These redox-active molecules mediate oxidative modifications of critical biomacromolecules—including proteins, lipids, and nucleic acids—resulting in cellular dysfunction and the pathogenesis of diverse disorders such as malignancies, cardiovascular diseases, and neurodegenerative conditions[[3], [4], [5], [6]]. As a hallmark neurodegenerative condition, Alzheimer's Disease (AD) manifests marked oxidative damage through three pathological hallmarks: amyloid-beta aggregation, compromised mitochondrial integrity, and deterioration of synaptic connectivity[7,8]. Compelling evidence indicates that ROS overproduction exacerbates Aβ oligomerization, lipid peroxidation, and neuronal apoptosis, thereby accelerating cognitive decline in AD[7,9,10]. Targeting oxidative stress with neuroprotective agents that preserve synaptic plasticity is therefore a critical goal in developing AD therapies[11,12]. Consequently, identifying natural antioxidants capable of mitigating oxidative damage and modulating related signaling pathways has emerged as a promising strategy for neuroprotection.

*Brassica rapa L.* (BR), a two-year-old plant belonging to the Brassicaceae family, is extensively grown in the Xinjiang region of China. It is valued for its bioactive phytochemicals, including flavonoids, glucosinolates, and phenolic acids, which contribute to its redox-modulating capacity[[13], [14], [15]]. Recent studies have demonstrated that *Brassica rapa L.* ethanolic extract (BREE)-derived ethyl acetate-soluble phytocomplex exhibits superior free radical scavenging activity, as evidenced by dose-dependent reductions in intracellular ROS levels and enhanced enzymatic antioxidant defenses (e.g., SOD as well as GSH-Px) in H_2_O_2_-induced PC12 cell models[16]. Furthermore, BREE-Ea significantly attenuates lipid peroxidation and lactic dehydrogenase (LDH) leakage, indicating its potential to preserve membrane integrity under oxidative stress[14,16]. Despite emerging evidence supporting BR's multifunctional bioactivities—such as anti-inflammatory and anti-hypoxic effects—systematic investigations into its molecular mechanisms of neuroprotection, particularly its modulation of mitochondrial dysfunction and apoptotic pathways, remain underexplored[17]. Elucidating these mechanisms could advance BR-derived nutraceuticals as therapeutic agents for neurodegenerative disorders linked to oxidative imbalance. In this context, in silico approaches, such as molecular docking, provide a powerful tool for preliminary screening of bioactive compounds and predicting their interactions with key neuroprotective targets, thereby guiding subsequent experimental validation[10,17].

PC-12 cells, a rat adrenal medulla-derived neuronal cell line, are extensively utilized in neurobiological and pharmacological studies due to their unique ability to differentiate into neuron-like phenotypes under specific conditions[18]. PC-12 cells, a well-established model for studying protein aggregation and neuronal apoptosis, have provided critical insights into the cellular processes contributing to AD, an age-associated encephalopathy with progressive synaptic failure, and related neurodegenerative disorders[[19], [20], [21]]. In experimental settings, H_2_O_2_-induced oxidative stress in PC-12 cells effectively recapitulates key pathological features of neurodegenerative diseases, including mitochondrial dysfunction, ROS overproduction, and neuronal apoptosis[16,20]. This model provides a robust platform for screening and evaluating the neuroprotective efficacy of antioxidants, enabling researchers to explore potential therapeutic strategies for mitigating oxidative damage in neuronal cells[16,18,19,22]. When combined with in silico techniques, this cellular model allows for a more efficient and mechanistic-driven discovery of candidate compounds, bridging computational predictions with biological validation.

Although the various biological activities of BR have attracted significant attention, research on its specific role in neurodegenerative diseases remains relatively scarce[16,[23], [24], [25]]. This study systematically evaluated *Brassica rapa L.* of ethanol extract's (BREE) antioxidant capacity and its cellular repair capability in PC-12 cells following oxidative injury. By integrating LC-MS-based metabolite profiling, targeted molecular docking (to prioritize key bioactive constituents and predict their protein targets), and transcriptomic analysis, we elucidated BREE's neuroprotective mechanisms and associated signaling pathways in oxidative stress-induced injury models. This multi-omics approach highlights the synergy between in silico screening and experimental validation in accelerating the discovery of plant-based neuroprotective agents. The findings provide mechanistic insights into BREE's therapeutic potential for neurodegenerative diseases and establish a framework for its application in functional food development.

## 2 Materials and methods

### 2.1 Materials and reagents

Fresh shade-dried slices of *Brassica rapa L.* (BR; supplier: Xinjiang Alinur Qiamagu Biotechnology Co., Ltd.) were purchased from Xinjiang, China, ground to pass through a 50-mesh sieve, and stored in self-sealing bags. hydrogen peroxide (≥30%, H_2_O_2_), ferric chloride (≥98%, FeCl_3_), potassium ferricyanide (≥99%, K_3_[Fe(CN)_6_]), trichloroacetic acid (≥99.5%, TCA), phosphate-buffered saline (PBS), salicylic acid (≥99%), Ascorbic acid (≥99%, Vc) and 2,2-diphenyl-1-picrylhydrazyl (≥95%, DPPH) from Aladdin Reagent Co., Ltd. (Shanghai, China). Cell culture supplements, including fetal bovine serum and horse serum, were sourced from Sangong Bioengineering Co., Ltd. (Shanghai, China). Sodium pyruvate (≥99%), RPMI 1640 medium, trypsin-EDTA (0.25%), and 96-well plates were obtained from Shanghai Taitan Technology Co. Cell Counting Kit-8 (CCK-8), commercially available from Shanghai Biyuntian Biotechnology Co., and undifferentiated PC12 cells were provided by the Chinese Academy of Sciences (Beijing, China).

### 2.2 Plant material and sample preparation

Fresh dried slices of BR were purchased from Xinjiang Alinur Qiamagu Biotechnology Co., Ltd., ground to pass through a 50-mesh sieve, and stored in self-sealing bags. For extraction, 20 g of accurately weighed BR powder was subjected to ultrasonic-assisted extraction (50 °C, 30 min, 300 W) using 80% ethanol (1:20 w/v) in a 60 °C water bath—conditions optimized in other prior study to maximize flavonoid yield while minimizing thermal degradation[16]. The residue was re-extracted twice under identical conditions. After filtration, the combined filtrates were concentrated via rotary evaporation to yield the BREE.

### 2.3 Liquid chromatography-mass spectrometry (LC-MS) for molecular characterization

Metabolite extraction was performed by adding prechilled extraction solvent (80% methanol containing 0.1% formic acid) to samples, followed by 5 min incubation on ice to minimize enzymatic degradation. Phase separation was achieved via centrifugation at 15,000×*g* for 20 min at 4 °C, with the supernatant collected for subsequent processing. To optimize analyte solubility and chromatographic performance, the supernatant was diluted to 53% methanol using LC-MS-grade water (Fisher Scientific), followed by re-centrifugation under identical conditions and 0.22 μm PVDF membrane filtration (Millipore) to remove particulate matter before instrumental analysis.

Metabolite separation and detection were performed using a Vanquish UHPLC system (Thermo Fisher Scientific) interfaced with a Q Exactive Plus Orbitrap mass spectrometer. Chromatographic execution utilized an ACQUITY UPLC HSS T3 column (1.7 μm particle size, 2.1 mm × 100 mm dimensions), with the column temperature controlled at 45 °C via a dedicated column oven. The mobile phase comprised phase A (water with 0.1% formic acid) and phase B (acetonitrile with 0.1% formic acid). A gradient program was applied, starting at 99:1 (A: B) and progressing to 0:90 (A: B) over 12 min, followed by a 1-min isocratic phase at 0:90 (A: B) to ensure full elution. Flow rate was regulated at 0.4 mL/min throughout the gradient to maintain system stability.

Mass spectrometric data acquisition was performed via intelligent precursor selection (DDA) in negative ionization mode with HESI source. Full-scan MS spectra (70–1000 *m*/*z*) were acquired at 70,000 resolution (AGC target: 1 × 10^6^, injection duration: 100 ms), followed by five targeted MS/MS events at 17,500 resolution (AGC target: 5 × 10^5^, injection duration: 50 ms). Fragmentation was induced through HCD with dual NCE settings (15% and 30%). Ion source parameters included spray voltage: 2.8 kV, capillary temperature: 320 °C, S-lens RF voltage: 50 V, with high-purity nitrogen (99.999%) employed as collision gas.

Data processing was conducted using Xcalibur 3.0 software, incorporating peak alignment algorithms, signal detection modules, and quantification pipelines with normalization to total ion current. Structural elucidation integrated isotopic distributions, molecular ion clusters, and diagnostic fragment ions. Metabolite annotation leveraged cross-referencing with mzCloud, mzVault, and MassList databases, enabling comprehensive structural characterization and semi-quantitative metrics.

### 2.4 Measurement of the antioxidant power of BREE

#### 2.4.1 Free radical scavenging assay (DPPH)

The DPPH radical scavenging capacity of BREE was evaluated following an optimized protocol adapted from established methodologies[26]. Briefly, a DPPH stock solution (0.2 mmol/mL) was freshly prepared by dissolving 0.789 mg of DPPH (≥95%, Aladdin) in 10 mL of 95% ethanol (v/v), followed by protection from light storage at 4 °C for stability. BREE samples were dissolved in 60% ethanol (1 g/100 mL) and serially diluted to final concentrations of 0–10 mg/mL. Vc (≥99%, Aladdin), prepared under the same conditions, served as the positive control.

For the assay, 1 mL of sample or control was mixed with 1 mL of DPPH solution in an amber vial, vortexed the mixture for 10 s, and allowed to incubate for 30 min at 25 °C protected from ambient illumination. Quantitative analysis was conducted at 517 nm wavelength with a UV-visible spectroscopy platform (Shimadzu, UVPC2450), with triplicate readings per sample. The formula for free radical scavenging efficiency (%) was calculated as:

(1) $$\text{Scavenging}\;\text{activity}\;\left(\%\right)=\left[1-\left(A_{\text{sample}}-A_{\text{sample}\;\text{control}}\right)/A_{\text{blank}}\right]\times 100$$

Where A _sample_ = absorbance_517_ of sample, A _sample control_ = absorbance_517_ for sample control (without DPPH), and A _blank_ = absorbance_517_ of blank solution(without sample). This method was repeated for all sample concentrations, and the results were used to calculate the IC50 value.

#### 2.4.2 Hydroxyl radical (·OH) scavenging efficiency

The hydroxyl radical (·OH) scavenging efficiency of BREE was evaluated by a Fenton reaction-based method with salicylate hydroxylation as the detection principle[26]. Briefly, hydroxyl radicals were generated by mixing 9 mM FeSO_4_(≥98%, Aladdin) and 0.3% H_2_O_2_ (≥30%, Sigma-Aldrich) in the presence of 5 mM salicylic acid dissolved in ethanol (≥99%, Macklin). BREE samples were dissolved in 60% ethanol (1 g/100 mL) and serially diluted to final levels from 0 to 10 mg/mL in increments of 2 mg/mL. Vc (≥99%, Aladdin) served as the positive control and was prepared identically.

For the assay, reaction mixtures containing BREE samples (1 mL), FeSO_4_ (1 mL), H_2_O_2_ (1 mL), and salicylic acid (0.5 mL) underwent thermal equilibration under standard culture conditions (37 °C, 30 min). The total volume was brought to 5 mL by adding distilled water, and the absorbance at 510 nm was recorded using a UVPC2450 spectrophotometer with triplicate determinations. The scavenging activity (%) was calculated as:

(2) $${OH}^{-}\;\text{radical}\;\text{scavenging}\;\text{rate}\;\left(\%\right)=\left[1-\left(A_{\text{sample}}-A_{\text{sample}\;\text{control}}\right)/A_{\text{blank}}\;\right]\times 100$$

Where A _sample_ = absorbance_510_ of the sample (salicylic acid), A _sample control_ = absorbance_510_ of the sample (without salicylic acid), and A _blank_ = absorbance_510_ of the blank (H_2_O_2_ + FeSO_4_ without sample). This method was repeated for all sample concentrations, and the results were used to calculate the IC50 value.

#### 2.4.3 Quantitative assessment of reductive capacity

The electron donation capability of BREE was assessed using a modified ferric ion reduction assay[26]. BREE (1 g) was dissolved in 60% ethanol (100 mL) and serially diluted to final levels from 0 to 10 mg/mL in increments of 2 mg/mL. Reaction mixtures containing 1 mL sample, each containing 2.5 mL of 0.1 M PBS (pH 6.6) and an equivalent volume of 1% K_3_[Fe(CN)_6_] (w/v) were incubated at 50 °C for 20 min. The reaction was halted by supplementing 2.5 mL of 10% TCA (w/v). Subsequently, 2.5 mL of the mixture was mixed with 2.5 mL ultrapure water and 0.5 mL of 0.1% FeCl_3_ (w/v), followed by 30-min chromogenic incubation at 25 °C. Absorbance measurements were performed at 700 nm on a UVPC2450 spectrophotometer, with triplicate determinations for each concentration.

### 2.5 Cell culture

PC-12 cells (obtained from the CAS Cell Repository) were maintained in RPMI-1640 basal nutrient solution enriched with heat-inactivated equine serum (10% v/v), FBS (5% v/v), sodium pyruvate (1 mM final concentration), and a dual-antibiotic system (penicillin-streptomycin cocktail: 100 U/mL and 100 μg/mL, respectively). Cell cultures were incubated in a controlled environment (37 °C, 5% CO_2_ saturation, 95% humidity). Subculturing was performed every 48 h using 0.25% trypsin-EDTA, and experiments exclusively used cells harvested during exponential proliferation (80–90% confluency)[27].

### 2.6 CCK-8 assay

Cell viability was measured using the CCK-8 assay.[28,29]. Logarithmic-phase PC-12 cells were distributed across 96-microwell plates (cell density: 5 × 10^4^ cells/mL) and cultured for a 24-h period in RPMI-1640 basal medium supplemented with 10% heat-inactivated equine serum to ensure adhesion. After aspirating the medium, a 100 μL aliquot of CCK-8 solution (prepared by diluting CCK-8 reagent to 10% in serum-free RPMI 1640) was administered to each well. Following a 1-2 h culture period at 37 °C/5% CO_2_, optical density (OD) was recorded at 450 nm with a BioTek Epoch microplate reader. Viability (%) was calculated relative to untreated controls using the formula:

(3) $$Viability\left(\%\right)=\left[\left(A_{\text{sample}}-A_{\text{blank}}\right)/\left(A_{\text{comtrol}}-A_{\text{blank}}\right)\right]\times 100$$

Where A _blank_ represents wells containing CCK-8 solution without cells. Triplicate measurements were performed for each experimental condition.

### 2.7 Modeling of H_2_O_2_-induced damage in PC-12 cells

PC-12 cells were resuspended in complete medium to 5 × 10^5^ cells/mL after trypsinization. A 100 μL aliquot of the suspension (5 × 10^4^ cells/well) was dispensed into 96-well plates, with peripheral wells filled with sterile PBS to minimize evaporation-induced artifacts. Triplicate wells were designated for each experimental condition. Cell cultures were kept in a 37 °C/5% CO_2_ environment for 24 h to ensure adherence. Subsequently, adherent cultures were exposed to H_2_O_2_ at concentrations ranging from 10 to 300 μM in serum-free RPMI 1640 medium for 6 or 12 h. At each time point, the medium containing H_2_O_2_ was removed and substituted with 100 μL of a 10% CCK-8 solution (prepared by diluting CCK-8 reagent in serum-free RPMI 1640). Following 1–2 h incubation under standard culture conditions, OD was recorded at 450 nm with a BioTek Epoch microplate reader.

### 2.8 Effect of BREE on PC-12 cell viability

PC-12 cells were resuspended in complete medium to 5 × 10^5^ cells/mL after trypsinization. A 100 μL aliquot of the suspension (5 × 10^4^ cells/well) was dispensed into 96-well plates, with peripheral wells filled with sterile PBS to mitigate evaporation artifacts. Triplicate wells were assigned for each experimental group. After a 2-h equilibration phase in a humidified 5% CO_2_ incubator at 37 °C, cell cultures received BREE exposure (0–250 μg/mL) for 6 h. At designated time points, the medium was aspirated, and Following a 1-h culture period at 37 °C/5% CO_2_, OD was recorded at 450 nm with a BioTek Epoch microplate reader.

### 2.9 Repairing effects of BREE on H_2_O_2_-injured PC-12 cells

PC-12 cells were resuspended in complete medium to 5 × 10^5^ cells/mL after trypsinization. An aliquot(100 μL) of the cell suspension (5 × 10^4^ cells/well) was dispensed into 96-well plates, with peripheral wells filled with sterile PBS to minimize evaporation artifacts. Triplicate wells were designated for each experimental group. After a 24-h equilibration phase under standard culture conditions (37 °C, 5% CO_2_), Cultures were subjected to oxidative challenge via 200 μM H_2_O_2_ dissolved in serum-deprived RPMI-1640 over a 12-h period to model oxidative stress. Post-treatment, the H_2_O_2_-containing medium was aspirated, and cells were incubated with BREE (0–250 μg/L) for 6, 12, or 24 h. At predetermined intervals, the BREE-containing medium was aspirated and replaced with 100 μL of 10% CCK-8 solution (diluted in serum-free RPMI 1640). After incubating for 1–2 h under standard conditions, absorbance values at 450 nm were acquired using a BioTek Epoch microplate reader.

### 2.10 Molecular docking

Based on the LC-MS analysis results and the observed repairing effects of BREE on H_2_O_2_-injured PC-12 cells, Molecular docking was performed to investigate the interactions between antioxidant components from BREE and key proteins involved in neurorepair pathways in PC-12 cells. Using a binding energy threshold of < −7.0 kcal/mol for screening, three representative flavonols—isorhamnetin, kaempferol, and quercetin—were selected along with four critical proteins implicated in neural repair mechanisms: protein kinase B (Akt)[30], phosphatase and tensin homolog (PTEN)[28,29], Trk system potassium ion transporter (Trk)[27], and cyclins. The 3D structures of target proteins were retrieved from the PDB database (Akt: PDB ID 8QAT; PTEN: PDB ID 3AWG; TrkA: PDB ID 4RO0; Cyclin B1: PDB ID 2B9R) via UniProt, while the 3D coordinates of flavonols (isorhamnetin: PubChem CID 5281654; kaempferol: PubChem CID 5280863; quercetin: PubChem CID 5280343) were obtained from PubChem. Ligand structures were converted from SDF to PDBQT format using Open Babel (v3.0.0) with energy minimization to ensure conformational accuracy.

AutoDock Vina (v1.1.2) was employed to perform molecular docking, with grid parameters optimized to encompass the active sites of each protein. For example, Grid box dimensions for each protein were optimized to encompass their active sites: Akt: 20 × 20 × 20 Å^3^(centered at residues 296–440); PTEN: 18 × 18 × 18 Å^3^(centered at residues 122–131); TrkA: 22 × 22 × 22 Å^3^(centered at residues 345–354); Cyclin B1: 20 × 20 × 20 Å^3^(centered at residues 145–154) [30,31]. Docking simulations were conducted with 50 independent genetic algorithm runs, and the lowest-energy binding poses were selected for analysis. Key interaction parameters, including hydrogen bond lengths (<3.5 Å), hydrophobic contacts (>4 interactions), and binding affinities (<−7.0 kcal/mol), were used to evaluate ligand–protein compatibility. Post-docking analysis was performed with PyMOL (v2.5) to visualize hydrogen bonds, π–π stacking, and electrostatic interactions. 2D interaction maps for the top binding poses (e.g., quercetin-Akt) were generated using PyMOL.

### 2.11 Transcriptome analysis

To elucidate the mechanisms underlying BREE-mediated repair of injured PC-12 cells and investigate the functional relevance of molecular docking predictions, transcriptomic profiling was performed. Logarithmic-phase PC-12 cells were trypsinized and resuspended in RPMI 1640 complete medium adjusted to 5 × 10^6^ cells/mL. A cell suspension (2.5 × 10^6^ cells per well) was plated in 6-multiwell dishes (1 mL/well), with subsequent 24 h stabilization in a normoxic environment (37 °C, 5% CO_2_). To induce oxidative stress, cultures were subjected to 200 μM H_2_O_2_ challenge in the serum-deprived medium for 12 h. After treatment, the medium containing H_2_O_2_ was discarded, followed by the addition of 150 μg/L BREE to the cultures for 6 or 12 h. After sequential rinsing with ice-cold PBS (pH 7.4), cellular detachment was achieved through trypsin treatment. The reaction was scavenged with complete medium, and cell pellets were obtained by centrifugation (5 min, 300×*g*, 4 °C). Following two PBS rinses, the pellets were subjected to TRIzol™ reagent (1 mL per 5 × 10^6^ cells) via vigorous pipetting. Lysates were transferred to pre-chilled RNase-free microtubes, flash-frozen in liquid nitrogen (30 min), and maintained at −80 °C until transcriptome analysis.

### 2.12 Statistical analysis

Results are presented as mean ± standard deviation (SD) from three independent replicates. Group comparisons were analyzed via one-way ANOVA (SPSS 26.0) with Tukey's post hoc test, and statistical significance was established at p < 0.05.

## 3 Results and discussion

### 3.1 Purification and identification of BREE

Building upon our previous investigations[32], we optimized the identification of bioactive constituents in BREE through LC-MS profiling (Table 1). The analysis revealed the presence of flavonols, organosulfur compounds, heterocyclic compounds, terpenoids, amino acid derivatives, natural products, and antibiotics. These compounds demonstrated robust antioxidant properties, with quercetin, kaempferol, and isorhamnetin—three flavonol subclasses—exhibiting particularly pronounced redox-regulating potential.

**Table 1:** Analysis of the antioxidant-active compounds of BREE by LC-MS.

Formula	Molecular weight	*m*/*z*	*Rt (min)*	Name	Category	Functions	Ref.
C_15_H_10_O_7_	302.25465	301.04136	15.184	Quercetin	Flavonols	Antioxidant, anti-inflammatory, anticancer	[33]
C_15_H_10_O_6_	286.25566	285.01034	16.327	Kaempferol	Flavonols	Antioxidant, anti-inflammatory, anticancer	[34]
C_16_H_12_O_7_	316.27813	300.01894	3.428	Isorhamnetin	Flavonols	Antioxidant, anti-inflammatory, cardioprotective, anticancer	[35]
C_11_H_13_NS	191.07693	190.06962	1.245	(4-Isothiocyanatobutyl)benzene	Organosulfur Compounds	Activation of the Keap1-Nrf2 signaling pathway and modulation of antioxidant defense systems	[36]
C_5_H_9_NS	115.04562	116.0529	3.386	Butyl isothiocyanate	Organosulfur Compounds	Exhibiting potent antioxidant activity and inducing the expression of phase II detoxification enzymes	[37]
C_11_H_12_N_2_O_2_S	236.06152	237.0688	4.991	Zileuton	Organosulfur Compounds	Inhibition of leukotriene biosynthesis and attenuation of inflammatory responses	[38]
C_8_H_10_N_4_O_2_	194.0801	195.08738	4.924	Caffeine	Heterocyclic Compounds	Neuroexcitatory effects coupled with antioxidant activity and free radical scavenging capacity	[39]
C_13_H_20_O	192.15114	193.15843	9.295	Alpha-Ionone	Terpenoids	Antioxidant-containing aromatic compounds or fragrance agents with redox-modulating properties	[40]
C_10_H_15_NO_6_	245.0896	246.09688	4.455	MYCOSPORINE GLYCINE	Amino Acids and Derivatives	UV-absorbing antioxidant barriers with photoprotective efficacy	[41]
C_10_H_16_N_2_O_3_	212.11594	213.12322	2.489	Staphyloamide B	Natural Products and Antibiotics	Exhibiting anti-inflammatory and antioxidant activities with reinforcement of antioxidant defense barriers	[42]

### 3.2 Antioxidant capacity of BREE

The antioxidant capacity of BREE was comprehensively evaluated using DPPH and hydroxyl radical scavenging assays, along with a reducing power assessment. The IC_50_ values, representing the concentration of the extract required to achieve a 50% radical scavenging effect, were used in the first two experiments. The results were compared with Vc (positive control). BREE at different concentrations (0–10 mg/mL) demonstrated good potential in scavenging radicals and reducing oxidative stress.

BREE's antioxidant activity is multifaceted: (1) DPPH scavenging targets lipid-soluble radicals, which are involved in lipid peroxidation; (2) Hydroxyl radical scavenging targets water-soluble radicals, which damage DNA and proteins; (3) Reducing power donates electrons to neutralize oxidants, preventing oxidative chain reactions. These combined effects mitigate oxidative stress, which is critical for protecting PC-12 cells from H2O2-induced damage.

#### 3.2.1 DPPH radical scavenging activity

**Fig. 1:**
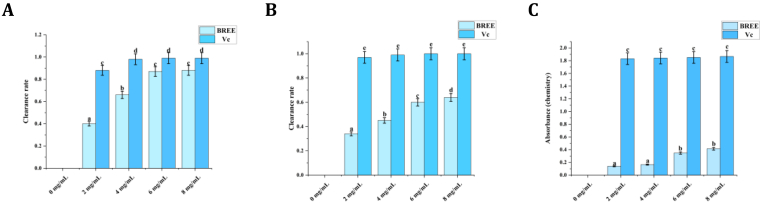
Antioxidant profiling of BREE through radical scavenging and redox-modulating assays. (A) DPPH• scavenging capacity. (B) Hydroxyl radical (•OH) elimination efficiency. (C) FRAP values expressed as Fe^2+^ equivalents (μM). Data are shown as mean ± SD (n = 3 replicates). Groups not sharing the same lowercase superscript differ significantly (p < 0.05, one-way ANOVA with Tukey's HSD test).

As shown in Fig. 1A, BREE demonstrated concentration-dependent DPPH radical scavenging activity across the 0–10 mg/mL test range, with scavenging rates increasing from 40% at 2 mg/mL to 88% at 10 mg/mL. The scavenging efficacy paralleled that of the positive control (ascorbic acid, Vc) at equivalent concentrations, with an IC_50_ value of 2.66 mg/mL (nonlinear regression analysis)—quantitatively validating its strong radical-quenching potential. The observed decline in 517 nm absorbance reflects BREE's capacity to neutralize DPPH• through electron transfer/hydrogen atom donation processes, a mechanism aligned with established antioxidant paradigms[16].

#### 3.2.2 Hydroxyl radical scavenging activity

Hydroxyl radicals (·OH), recognized as the most aggressive ROS due to their extreme oxidative reactivity, are implicated in extensive oxidative damage to biomolecules, thereby promoting cellular dysfunction and pathological conditions associated with oxidative stress. As presented in Fig. 1B, BREE demonstrated dose-dependent ·OH radical neutralization, achieving 72% scavenging efficiency at 10 mg/mL. The antioxidant profile of Vc exhibited comparable concentration-response dynamics under standardized assay conditions. Notably, BREE revealed rapid radical attenuation within the 0–6 mg/mL range, with scavenging kinetics approaching saturation at elevated concentrations. The calculated IC_50_ value of 3.32 mg/mL (nonlinear regression analysis) quantitatively underscores BREE's capacity to counteract ·OH-induced oxidative stress. The underlying mechanism likely involves hydrogen atom transfer (HAT) and single-electron transfer (SET) pathways, which destabilize ·OH and interrupt oxidative chain propagation in lipid and protein systems. Comparative assessment with reported natural antioxidants confirms BREE's efficacy within established antioxidant benchmarks, supporting its potential as a redox-modulating agent[16].

#### 3.2.3 Reducing power

The ferric-reducing antioxidant power (FRAP) assay demonstrated BREE's concentration-dependent electron transfer capacity, with a linear increase in 700 nm absorbance observed across the 0–10 mg/mL range (Fig. 1C). This quantitative correlation reflects BREE's ability to reduce Fe^3+^ to Fe^2+^ through SET pathways, thereby modulating redox-active metal ions implicated in ROS generation. The antioxidant activity profile correlates with the phytochemical composition, as phenolic and flavonoid metabolites constitute key functional moieties mediating plant-derived oxidative stress defense. The elevated polyphenolic content in BREE likely underpins its radical-quenching efficacy via synergistic mechanisms involving direct scavenging and metal chelation. These observations corroborate established literature on natural antioxidants, where polyphenolic architectures regulate redox homeostasis through dual-action mechanisms[16].

### 3.3 PC-12 cell oxidative damage model

**Fig. 2:**
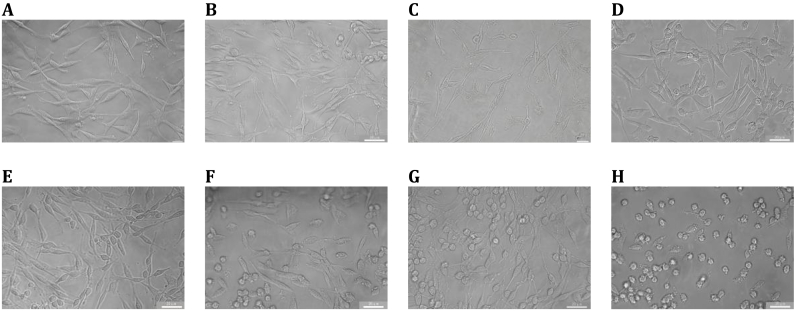
H_2_O_2_-induced morphological alterations in PC-12 neuronal cells. (A) Morphological features of untreated controls. (B–H) Progressive cellular damage following 12-h exposure to escalating H_2_O_2_ concentrations (10,25,50,100,200,300,600 μmol/L). Data are expressed as mean ± SD (n = 3 replicates). Groups sharing distinct lowercase superscripts differ significantly (one-way ANOVA with Tukey's HSD test, α = 0.05).

The H_2_O_2_-induced oxidative stress model exhibited concentration- and time-dependent cytotoxicity in PC-12 cells, as quantified by CCK-8 assays (Fig. 5A). Cell viability negatively correlated with both H_2_O_2_ exposure duration (6–12 h) and concentration (0–300 μmol/L), with viability dropping from 159 ± 7.9% (25 μmol/L, 12 h) to 32.7 ± 1.6% (600 μmol/L, 12 h). Morphological changes, including cytoplasmic condensation and membrane blebbing (Fig. 2), corroborated dose-dependent cellular injury. Optimization identified 200 μmol/L H_2_O_2_ for 12 h as the sublethal paradigm (51.3 ± 1.6% survival), balancing oxidative damage induction with preservation of membrane integrity for repair studies.

Reaction kinetics analysis established 1 h incubation as optimal for CCK-8 signal stability, minimizing background interference while maintaining sensitivity. This model demonstrated robust reproducibility across triplicates and aligned with standardized neuronal injury protocols, effectively recapitulating neurodegenerative oxidative pathology without inducing necrotic endpoints. Subsequent antioxidant rescue assays validated its utility for mechanistic redox studies.

### 3.4 Effects of BREE on PC-12 cell viability

**Fig. 3:**
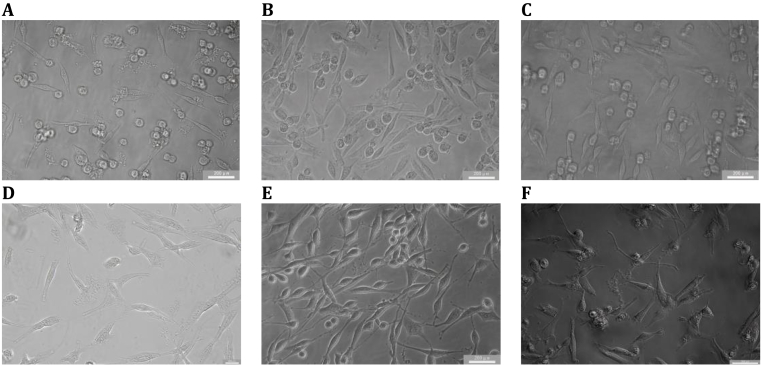
BREE exerts modulatory effects on morphological homeostasis in unchallenged PC-12 cells. (A) Unperturbed control morphology. (B–F) Cellular responses to 24-h exposure of BREE (50–250 μg/mL), demonstrating preserved viability (CCK-8 assay) and structural integrity across all concentrations. Statistical analysis revealed no significant intergroup variation (P > 0.05, one-way ANOVA with Tukey's HSD test). Data represent mean ± SD (n = 3 independent experiments). Lowercase superscripts indicate statistically distinct groups (α = 0.05).

The cytotoxic potential and neuroprotective capacity of BREE were assessed in PC-12 cells using the CCK-8 assay. As depicted in Fig. 3, Fig. 5B, BREE demonstrated no significant cytotoxicity across concentrations ranging from 0 to 250 μg/mL. Notably, BREE-treated cultures exhibited a modest viability enhancement relative to untreated controls (e.g., 134.4 ± 6.7% at 200 μg/mL versus 100% in controls, P > 0.05), coupled with preserved cellular morphology. These observations suggest a possible neurotrophic influence on neuronal viability under non-pathological conditions. BREE concentrations up to 250 μg/mL were non-toxic to PC-12 cells, with viability remaining >90% compared to untreated controls. This confirms the safety of the selected range for subsequent experiments.

### 3.5 Repairing effects on H_2_O_2_-Injured PC-12 cells

**Fig. 4:**
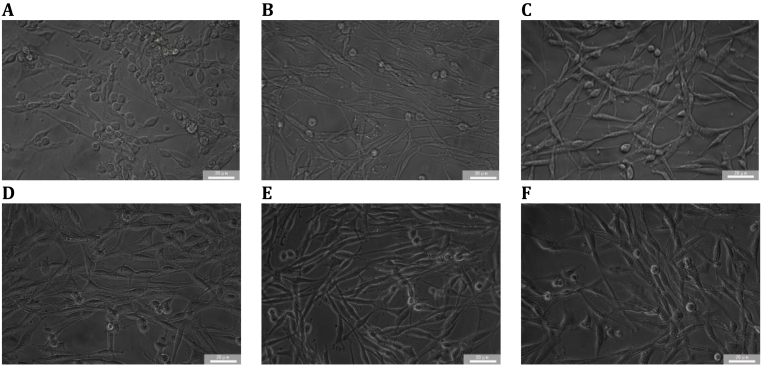
BREE-regulated morphological restoration in oxidatively damaged PC-12 cells. (A) Unperturbed morphology of sham-treated controls. (B–F) Dose-dependent restoration of cellular integrity after 6-h BREE intervention (50–250 μg/mL), with peak neuroprotective effects observed at 200 μg/mL. Morphological analysis revealed reduced cytoplasmic vacuolization and restored neurite integrity in BREE-treated cells. Data represent mean ± SD (n = 3 biological replicates). Lowercase superscripts indicate distinct clusters identified by one-way ANOVA with Tukey's HSD test (α = 0.05).

**Fig. 5:**
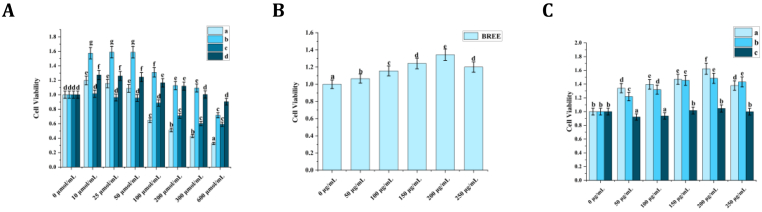
BREE modulates redox homeostasis in PC-12 cells. (A) PC-12 cell oxidative damage model, Conditions: a | 12-h H_2_O_2_ exposure + 1-h CCK-8 incubation, b | 12-h H_2_O_2_ exposure + 2-h CCK-8 incubation, c | 6-h H_2_O_2_ exposure + 1-h CCK-8 incubation, d | 6-h H_2_O_2_ exposure + 2-h CCK-8 incubation. (B) Effects of BREE on PC-12 Cell Viability. (C) Temporal dynamics of BREE repair capacity in H_2_O_2_-damaged PC-12 cells (6,12,24-h intervention window). Data represent mean ± SD (n = 3 biological replicates). Lowercase superscripts indicate distinct clusters identified by one-way ANOVA with Tukey's HSD test (α = 0.05).

As demonstrated in Fig. 4, Fig. 5C, In the neuronal PC-12 cell model pre-treated with H_2_O_2_ to induce oxidative injury, post-treatment administration of BREE exerted significant concentration-dependent reparative effects, effectively restoring cellular integrity and counteracting peroxide-mediated damage. Exposure to 200 μmol/L H_2_O_2_ for 12 h reduced cell viability to 52.3% ± 4.1% compared to untreated controls. Subsequent treatment with BREE (50–250 μg/mL) significantly reversed this damage, showing dose-responsive restoration that peaked at 200 μg/mL after 6 h (162.3% ± 6.9%, P < 0.001),12 h (148.3% ± 7.4%, P < 0.01) and 24 h (104.6% ± 5.2%, P < 0.001). Notably, the 6 h treatment group displayed superior cytoprotection relative to longer incubation periods, suggesting rapid ROS scavenger and mitochondrial functional rescue, consistent with its free radical scavenging capacity (Fig. 1A–C). where polyphenolic constituents mitigate oxidative cascades through enzymatic and non-enzymatic pathways. Collectively, BREE demonstrates potent reparative effects against neuronal oxidative damage, positioning it as a viable candidate for neuroprotective interventions.

### 3.6 Molecular docking

**Fig. 6:**
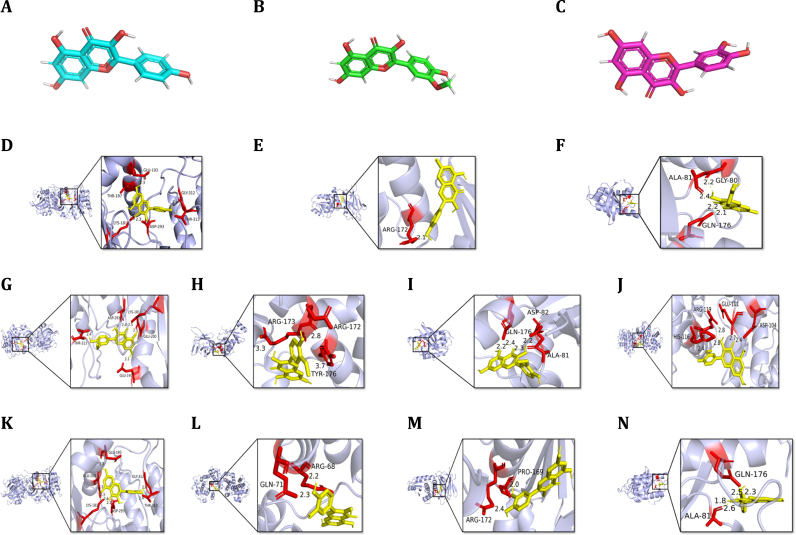
Molecular structures and binding modes of flavonols with key neuronal repair proteins. (A–C) Three-dimensional structures of flavonols: (A) Kaempferol, (B) Isorhamnetin, (C) Quercetin. (D–L) Molecular docking analysis of flavonol-protein interactions in injured PC-12 cells: Kaempferol: (D) Akt/PKB, (E) PTEN, (F) TrkA Isorhamnetin: (G) Akt/PKB, (H) PTEN, (I) TrkA, (J) Cyclin B1 Quercetin: (K) Akt/PKB, (L) PTEN, (M) TrkA, (N) Cyclin B1.

To elucidate BREE's mechanism of action in PC-12 cell repair, molecular docking was performed between three flavonol constituents (isorhamnetin, kaempferol, quercetin) (Table 1, Fig. 6A–C) and key regulators of the PI3K/Akt and Cell Cycle pathways: Akt, PTEN, Trk receptors, and cyclins. Computational analysis revealed high-affinity interactions across all ligand-protein pairs, with binding energies ranging from −7.021 to −8.762 kcal/mol and optimal "S-class" docking scores (Fig. 6D–N). Isorhamnetin demonstrated the strongest binding to Akt (−8.762 kcal/mol), forming critical hydrogen bonds with residues (e.g., ASP-82, GLN-176) alongside π–π stacking interactions with aromatic amino acids. Similar patterns were observed for kaempferol and quercetin, though quercetin exhibited superior Trk receptors affinity (−7.376 kcal/mol), engaging in dual hydrogen bonding with GLN-176 and ALA-81(Fig. 6I). Notably, all compounds maintained stable interactions with PTEN (binding energies: −7.35 to −7.479 kcal/mol), suggesting modulation of its phosphatase activity to counteract oxidative stress-induced pathway dysregulation.

Structural analysis highlighted distinct target preferences: isorhamnetin primarily engaged the PI3K/Akt axis through Akt-PTEN interactions, while quercetin showed enhanced promiscuity toward cyclins, aligning with its documented role in Cell Cycle pathways. Kaempferol displayed intermediate binding profiles, reinforcing the hypothesis that flavonol scaffolds exert pleiotropic effects via complementary pathway modulation. While Trk receptor binding affinities fell within a moderate range (−7.021 to −7.376 kcal/mol), sustained bidentate hydrogen bonding with Gln574 suggests modulation of neurotrophin signaling cascades. This structural interaction may complement antioxidant pathways through parallel neuroprotective mechanisms. These findings corroborate prior reports demonstrating flavonoids' dual capacity to regulate survival pathways and Cell Cycle pathways, providing a mechanistic basis for BREE's reparative effects in H_2_O_2_-injured PC-12 cells[43].

### 3.7 Transcriptome analysis of BREE effect on oxidative stress

**Fig. 7:**
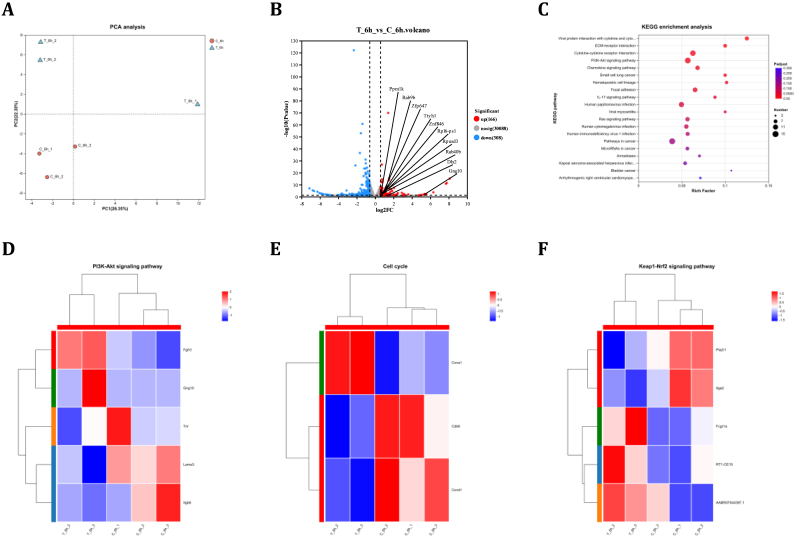
Transcriptomic Profiling of Post-Injury Repair Mechanisms in PC-12 Cells. (A) Principal component analysis (PCA) of transcriptomic data, distinguishing H_2_O_2_-injured (blue) and BREE-treated (red) groups. (B) Volcano plot of transcriptional variations. (C) KEGG analysis of the top 20 significantly enriched pathways, with PI3K-Akt signaling prominently identified. (D–F) Hierarchical clustering heatmaps of DEGs associated with Antioxidant Repair-related pathways. Data derived from RNA-seq analysis (n = 3 biological replicates).

Transcriptomic profiling was employed to dissect BREE's neuroprotective mechanisms against H_2_O_2_-induced PC-12 cell injury and validate computational docking predictions. Principal component analysis (PCA) demonstrated distinct transcriptomic patterns between BREE-treated (6h) and control groups (Fig. 7A). Application of |log_2_FC| > 0 and P < 0.05 thresholds identified 474 differentially expressed genes (DEGs), with 166 upregulated and 308 downregulated transcripts compared to untreated controls (Fig. 7B). KEGG pathway enrichment mapped these DEGs to 20 signaling pathways (Fig. 7C), while hierarchical clustering revealed dynamic expression profiles in antioxidant defense-related modules, specifically within PI3K/Akt, Cell Cycle pathways, and Keap1-Nrf2 signaling pathway (Fig. 7D–F).

Functional characterization demonstrated BREE's capacity to modulate oxidative stress responses via multiple mechanisms: transcriptional activation of neuroprotective genes (*Gng10, Ttyh1, Dlx2*), enhanced antioxidant enzyme expression through Keap1-Nrf2 signaling, and regulation of mitochondrial bioenergetics via PI3K/Akt and Cell Cycle pathways. These findings corroborate molecular docking predictions and in vitro validation data, demonstrating BREE's cytoprotective effects through coordinated antioxidant gene network modulation. The observed transcriptional reprogramming provides molecular evidence for BREE's dual functionality as both a nutraceutical agent and potential therapeutic for oxidative stress-associated neurological disorders.

## 4 Conclusions

In conclusion, this study demonstrates that BREE, derived from Xinjiang *BR*, effectively repairs oxidative damage in PC-12 neuronal cells following H_2_O_2_-induced injury through dual mechanisms of radical scavenging and redox signaling modulation. Molecular docking revealed direct interactions between BREE flavonoids (isorhamnetin, kaempferol, quercetin) and key antioxidant regulators Nrf2/Akt. Transcriptomic validation confirms the activation of PI3K/Akt and Keap1-Nrf2 signaling pathway, showing significant upregulation of neuroprotective genes (*Gng10, Ttyh1, Dlx2)* linked to mitochondrial bioenergetics and stress resilience. Functionally, BREE restores cell viability and preserves membrane integrity. BREE exhibits robust radical-scavenging capacity and ferric ion reduction ability, with polyphenolic constituents mechanistically coordinating both direct ROS neutralization and adaptive redox signaling reprogramming. These findings establish BREE as a dual-action neuroprotectant that first physically counteracts oxidative stress through electron transfer mechanisms, then epigenetically reinforces cellular stress defenses via Keap1-Nrf2 signaling pathway activation. The integrated multi-omics approach provides mechanistic evidence for developing BREE-based functional foods targeting age-related neurodegenerative conditions associated with oxidative imbalance.

## Funding statement

This study was supported by the Shanghai Agricultural Science and Technology Innovation Action Plan (2022; 22N31900200); Royal Society of New Zealand Catalyst Seeding Fund (CSG-UOA2108 and CSG-UOA2310).

## CRediT authorship contribution statement

**Wenyuan Wan:** Conceptualization, Data curation, Formal analysis, Methodology, Writing – original draft. **Yuntao Zhang:** Data curation, Investigation, Methodology, Writing – review & editing. **Xiaotong Yang:** Investigation, Methodology, Project administration, Writing – review & editing. **Jinyao Li:** Conceptualization, Methodology, Resources, Validation, Writing – review & editing. **Jun Lu:** Conceptualization, Formal analysis, Funding acquisition, Investigation, Resources, Supervision, Writing – review & editing. **Yu Zhao:** Conceptualization, Funding acquisition, Investigation, Project administration, Resources, Supervision, Writing – review & editing.

## Declaration of competing interest

No competing interests are declared by the authors.

## Data Availability

Data will be made available on request.

## Nomenclature

BR: *Brassica rapa* L.
BREE: *Brassica rapa L.* ethanolic extract
Vc: Vitamin C
AD: Alzheimer's Disease
LDH: lactic dehydrogenase
