# Supplementary material for: Targeted molecular docking-guided insights into the antioxidant reparative mechanisms of Brassica rapa L. extract after H2O2-induced neurotoxicity in PC-12 cells: Integration of LC-MS metabolomics and transcriptomic responses
Source: Biochem Biophys Rep. 2026 Mar 30;46:102566. doi: 10.1016/j.bbrep.2026.102566 (PMC13068608; doi:10.1016/j.bbrep.2026.102566)
Supplement: Multimedia component 1 [file mmc1.pdf]

outlook-icon Outlook

Decision on submission to Applied Food Research

From em.afres.0.9a0ed7.6a8b3eb6@editorialmanager.com
<em.afres.0.9a0ed7.6a8b3eb6@editorialmanager.com>
on behalf of
Applied Food Research <em@editorialmanager.com>

Date Sat 3/14/2026 10:53 AM

To Jun Lu <jun.lu@auckland.ac.nz>

Manuscript Number: AFRES-D-25-01824R1

Targeted molecular docking-guided insights into the antioxidant reparative mechanisms of Brassica rapa L. extract after H2O2-induced neurotoxicity in PC-12 cells: Integration of LC-MS metabolomics and transcriptomic responses

Dear Professor Lu,

Thank you for submitting your manuscript to Applied Food Research.

After careful evaluation, and following reviewer assessment, I regret to inform you that I am unable to accept your manuscript for publication in Applied Food Research. However, I believe the manuscript is suitable for publication in an alternative journal.

Based on my recommendation and the comments received, I expect that the Editor(s) of this alternative journal will accept your manuscript for publication without further review, provided you address any comments at the end of this email:

• Next Research, the peer-reviewed multidisciplinary journal in the Next family of journals, invites authors to publish original and scientifically rigorous research across all disciplines, accepting both theoretical and experimental work. As a broad-scope journal, our primary criterion for publication is the scientific accuracy and ethical integrity of the work, rather than its perceived impact. Submissions to Next Research are initially exclusively by transfer invitation; our dedicated team ensures a seamless and supportive publication experience from start to finish. [More information: https://www.sciencedirect.com/journal/next-research]
• Biochemistry and Biophysics Reports (CiteScore: 4.8; Impact Factor: 2.2; CAS Ranking: Tier 4) is an open access, online only, international journal which publishes original research in all aspects of Biochemistry, Biophysics and related areas like Molecular and Cell Biology. It has a high acceptance rate (42%) and the primary criteria for acceptance is that the work is original, scientifically and technically sound and provides valuable knowledge to life sciences research. As well as appearing in the Web of Science, all content published in the journal is indexed in PubMed Central, Scopus and the DOAJ. [More information: https://www.sciencedirect.com/journal/biochemistry-and-biophysics-reports]

If you agree with our recommendation, simply click Agree to Transfer by Jul 21, 2026. Please note that you will be given an opportunity to update your materials at the submission verification step, before delivery to our fellow Editors.

We appreciate you submitting your manuscript to Applied Food Research and thank you for giving us the opportunity to consider your work.

I understand that this second transfer offer may be frustrating and come as a disappointment, but I hope that one of the suggested journals could be suitable for your manuscript this time, and I hope this will help. Good luck with your new submission!

Kind regards,

Gabriela Marinho Righetto
Scientific Handling Editor
Applied Food Research

Additional Recommendations and Comments from the Scientific Handling Editor:

Thank you for submitting the revised version of your manuscript. As I had previously mentioned, we find that the manuscript currently do not align with the standards required for publication in Applied Food Research as it primarily focuses on biological activity and has limited emphasis on food processing and technology, which are the main interests of our journal.

Please note that our editorial assessment follows the publisher's guidelines to respect your journal choice and to ensure an impartial peer review process. Based on the reviewers' feedback and the journal's criteria for publication, we evaluate the suitability of the revised manuscript. If the manuscript is deemed suitable for publication but does not meet the specific editorial requirements of the current journal, we can offer to transfer it to other appropriate journals within our portfolio. This approach allows more authors to benefit from valuable feedback and revision opportunities, ultimately increasing their chances of successful publication either in the journal or through the transfer process. For further context on our approach, you may find the article Why Peer Review More? helpful: https://www.elsevier.com/connect/why-peer-review-more.

Minor comments:

Please display equations separately from your text, numbering them consecutively in the order they are referred to within your text. For more information, please check the Guide for Authors

Please ensure that the funding statement is presented separately from the acknowledgments section. According to our Guide for Authors, the acknowledgments should include any individuals who provided assistance during your research, such as help with language, writing, or proofreading. The funding information should be provided as a distinct statement, clearly outlining the sources of financial support for the study.

The Highlights section requires revision. The sentences are often incomplete; please revise to ensure they are concise, yet complete and clear, effectively highlighting the main contributions of your work.

Please revise the scale bars on your microscopy images to improve their visibility and ensure they are easily interpretable. Additionally, kindly provide the original images along with their metadata to facilitate review and verification of the microscopy data.

The contributions of several authors do not meet the authorship criteria outlined by ICMJE. To qualify as an author, someone must meet all four following criteria:
1. Substantial contributions to the conception or design of the work; or the acquisition, analysis, or interpretation of data for the work; AND
2. Drafting the work or reviewing it critically for important intellectual content; AND
3. Final approval of the version to be published; AND
4. Agreement to be accountable for all aspects of the work in ensuring that questions related to the accuracy or integrity of any part of the work are appropriately investigated and resolved.
Please either remove these authors from the author list (their contributions can be recognized in an "Acknowledgements" section instead), or confirm that these authors performed other contributions which would meet the criteria for authorship. Please explain in detail what each author's contributions are, describing exactly what each author did in this specific study/manuscript.
Please note that if you cannot adequately justify the presence of authors with appropriate contributions, these authors should be removed from the author list, or your submission cannot be considered for publication.
If you remove authors, please follow the guidelines described in our guide for authors page.

Reviewer Comments (if applicable):

Reviewer 1: Although some advanced computational approaches (such as molecular dynamics simulations) were not included in the study, the authors have carefully addressed the reviewers' comments by clearly justifying their methodology, providing the missing experimental details, and substantially improving the overall quality of the manuscript. These revisions have significantly enhanced the scientific rigor and reproducibility of the work. I appreciate the authors' diligent efforts during the revision process and recommend the manuscript for acceptance in its current form.

Reviewer 3: Targeted molecular docking-guided insights into the antioxidant reparative mechanisms of Brassica rapa L. extract after H2O2-induced neurotoxicity in PC-12 cells: Integration of LC-MS metabolomics and transcriptomic responses represent. The authors have been addressed properly and verifying that the final version is ready for publication
* The results and conclusion: compare the revised manuscript authors properly addressed all minor issues.
* Explanation of the antioxidant power of BREE: Explanation of antioxidant power of BREE very well.

More information and support

* When the transfer of your manuscript to the alternative journal is complete (usually within 48 hours) you will receive an email containing further instructions on how to complete your submission.

* You will be able to revise your manuscript prior to resubmission. When you submit your revised manuscript, please ensure you include a cover letter detailing all changes to the manuscript, as well as a "Response to Reviewers" containing all reviewer comments and a response to each comment.

* Once your submission is complete it will be considered by the Editor(s) of the alternative journal. While I cannot guarantee publication in the alternative journal, it is expected your manuscript will be accepted if the comments below are addressed.

You will find more information about Elsevier's Article Transfer Service here: www.elsevier.com/authors/article-transfer-service.

You will find information relevant for you as an author on Elsevier’s Author Hub: https://www.elsevier.com/authors

FAQ: How can I reset a forgotten password?
https://service.elsevier.com/app/answers/detail/a_id/28452/supporthub/publishing/
For further assistance, please visit our customer service site:
https://service.elsevier.com/app/home/supporthub/publishing/
Here you can search for solutions on a range of topics, find answers to frequently asked questions, and learn more about Editorial Manager via interactive tutorials. You can also talk 24/7 to our customer support team by phone and 24/7 by live chat and email

#AU_AFRES#

To ensure this email reaches the intended recipient, please do not delete the above code.

In compliance with data protection regulations, you may request that we remove your personal registration details at any time. (Remove my information/details). Please contact the publication office if you have any questions.
